# The influence of LPL S447X variants and obesity on lipid profile, oxidative stress, and the risk of T2DM: A case‐control study

**DOI:** 10.1002/hsr2.1031

**Published:** 2023-01-02

**Authors:** Gulshan Omar Ahmed, Zohreh Rahimi, Maryam Kohsari, Ebrahim Shakiba

**Affiliations:** ^1^ Department of Clinical Biochemistry Kermanshah University of Medical Sciences Kermanshah Iran; ^2^ Medical Biology Research Center, Kermanshah University of Medical Sciences Kermanshah Iran

**Keywords:** anthropometric parameters, lipid profile, LPL variants, obesity, oxidative stress parameters, type 2 diabetes mellitus

## Abstract

**Aims:**

The present study aimed to investigate the association between lipoprotein lipase (LPL) S447X polymorphism and type 2 diabetes mellitus (T2DM), obesity, lipid profile, and oxidative stress parameters in a population from the Kurdistan region of Iraq.

**Method:**

We studied 250 adults (51% female and 49% male) aged 45–65 years in four groups, obese and normal body mass index (BMI) diabetic patients versus healthy normal BMI and obese individuals as controls. Lipid profile and oxidative stress parameters were analyzed by colorimetric assay. The LPL S447X genotypes were detected by polymerase chain reaction (PCR)‐restriction fragment length polymorphism.

**Results:**

We found that the obese diabetic group had higher levels of triglycerides (TG), cholesterol, and low‐density lipoprotein‐cholesterol, and lower level of high‐density lipoprotein‐cholesterol than other groups. Obese diabetic patients had higher anthropometric indices than nonobese diabetic patients, obese and normal BMI controls. The levels of TG and total oxidative status (TOS) were significantly lower and higher, respectively, in normal BMI controls than in obese controls. Obese diabetic patients had a lower level of total antioxidant capacity than nondiabetic obese controls. The level of TOS was lower in nondiabetic controls compared to the patient groups. Obese diabetic patients had the highest TOS and malondialdehyde levels. The LPL SX genotype was associated with decreased the risk of T2DM by 79% (odds ratio [OR] = 0.21; 95% confidence interval [CI]: 0.05–0.81, *p* = 0.03). Also, the presence of this genotype reduced the risk of obesity by 39% (OR = 0.61; 95% CI: 0.07–4.90, *p* = 0.6). In all individuals, the presence of the SX genotype was associated with significantly lower levels of fasting blood sugar (FBS) and TOS.

**Conclusion:**

We report the influence of obesity on lipid profile in diabetic and nondiabetic individuals and the effect of LPL SX genotype on decreased risk of T2DM and reduced levels of FBS and TOS.

## 1 INTRODUCTION

Diabetes mellitus (DM), a complex metabolic disorder, over decades has become a significant subject of medical investigation. Type 2 diabetes mellitus (T2DM) along with T1DM are the leading causes of the burden of diabetes worldwide.^1^ The aetiological risk factors of T2DM include age, family history, ethnicity, diet quality, and metabolic disorders.^1^, ^2^

Dyslipidemia takes the main role in distributed vascular lesions, is more prevalent in diabetic patients, and places them at a remarkably higher risk for cardiovascular disease (CVD).^3^, ^4^ Dyslipidemia is characterized by abnormal levels (above the normal range) of one or more lipid profile parameters.^2^ Multiple investigations have demonstrated a correlation between dyslipidemia and the quality of T2DM control in patients.^5^ A Japanese cohort study on T2DM patients illustrated serum triglycerides (TG) as a much more influential factor than the A1_C_ hemoglobin for predicting CVD in DM patients, even in those with a higher level of blood glucose.^6^ Furthermore, dyslipidemia is associated with oxidative stress. Oxidative stress‐induced lipid peroxidation contributed to endothelial dysfunction, consequently causing the formation of atherosclerotic plaques and the incidence of diabetic nephropathy.^6^, ^7^

Lipoprotein lipase (LPL) is a glycoprotein enzyme located on chromosome 8.^8^ LPL enzyme has a known vital role in lipid metabolism via producing free fatty acids and monoacyl glycerol for tissue metabolic functions and energy storage by hydrolysis of chylomicrons and TG.^8^ Also, LPL possibly has a connection role in the uptake of lipoprotein by the cells and therefore is related to the regulation of plasma lipoproteins levels.^9^ More than 40 mutations have been detected in the locus of LPL gene in humans until now.^8^ In the LPL gene the C to G substitution (Ser^447^→Stop) produces the LPL protein with C‐terminal end truncation by two amino acids.^10^ LPL S447X polymorphism has been reported to be involved in the DM pathogenesis.^8^, ^10^ Studies have suggested that the LPL S447X polymorphism is associated with raising LPL protein secretion and plasma post‐heparin activity and also could decrease the plasma level of TG.^11^, ^12^

Since the exact mechanism of lipid metabolism dysfunction in diabetes is not fully understood and also the effect of ethnicity on the identification of genes study should not be ignored, the current study was performed to examine the possible association of LPL S447X variants with T2DM and obesity. Also, the effect of LPL S447X genotypes on the levels of oxidative stress, anthropometric and lipid profile parameters were investigated in a population from the Kurdistan of Iraq.

## 2 METHODS

### 2.1 Study design and population

The present case‐control study was carried out in Sulaimani, the Kurdistan region of Iraq. Data was obtained from the medical and paraclinical information of 250 adults from Sulaimani in the Kurdistan region of Iraq. All the participants were recruited from those who referred to the Sulaimani Endocrine and Diabetic Center, public health laboratory, and Sulaimani Shar Hospital from June to December 2021. The study included 129 (51%) females and 121 (49%) males aged 45–65 years. The study population was divided into four groups: obese diabetic patients (*n* = 69; 27.3%), normal BMI diabetic patients (*n* = 66; 26.1%), obese nondiabetic (*n* = 62; 24.5%), and normal BMI nondiabetic patients (*n* = 56; 22.1%). Nondiabetic individuals were considered as controls.

### 2.2 Study definition

T2DM was recognized based on the fasting blood glucose (FBS) over 126 mg/dl, and an oral glucose tolerance test (more than 200 mg/dl serum glucose level after 2 h of 75 g oral glucose consumption) twice according to the World Health Organization (WHO) guidelines.^1^

### 2.3 Inclusion and exclusion criteria

Individuals aged 45–65 years with T2DM and nondiabetic people with and without obesity were included in the investigation. People with underlying disorders that may affect the study results, such as T1DM, thyroid, and renal dysfunction, CVDs, and malignancy, and people who received medication for dyslipidemia and also pregnant women, were excluded from the investigation.

Obesity was defined as the body mass index (BMI) above 30 kg/m^2^. BMI was calculated by dividing weight (kg) by the square of height (m^2^). Also, all the anthropometric indices such as waist circumference, hip circumference, and wrist circumference were measured by a 0.1 cm accuracy stadiometer.

### 2.4 Sample collection and measurements

Blood samples were drawn after an overnight fasting. A total of 5 ml of venous blood was obtained from each participant. For DNA extraction, falcon tubes containing ethylenediaminetetraacetic acid were used to store whole blood samples (a volume of 2 ml). In the remaining samples serum was separated for biochemical analysis. FBS was detected using the standard enzymatic method by an automated chemical analyzer (Cobas c311). Lipid profile, including total cholesterol (Chol), TG, high‐density lipoprotein‐cholesterol (HDL‐C), and low‐density lipoprotein‐cholesterol (LDL‐C), were measured with an enzymatic colorimetric assay. Total antioxidant capacity (TAC) was analyzed by a colorimetric assay kit (Kiazist Co.). In this method a color solution is produced by a chromogen reagent affected by the reduction of Cu^2+^ to Cu^+^ by antioxidants at the wavelength of 450 nm, which was detected via a spectrophotometer. Total oxidative status (TOS) was also measured by a colorimetric method kit (Kiazist Co.). In this method, the ferrous ion (Fe^2+^) in the presence of oxidants is converted to the ferric ion (Fe^3+^) that, in the presence of the chromogen, produces a colored solution with an absorbance at the wavelength of 550–580 nm. The malondialdehyde (MDA) concentration was determined colorimetrically using thio‐barbituric acid method.^13^

### 2.5 Genotyping

The phenol‐chloroform method was used for DNA extraction.^14^ The variants of the LPL S447X were detected via polymerase chain reaction (PCR)‐restriction fragment length polymorphism. The PCR was performed using 20 pmol of each primer (forward primer: 5′‐TACACTAGCAATGTCTAGGTGA‐3′ and the reverse primer: 5′‐TCAGCTTTAGCCCAGAATGC‐3′). The 488‐bp PCR product was digested with Mnl1 restriction enzyme as previously described (10).

### 2.6 Statistical analysis

Data analysis was carried out by SPSS software (SPSS Inc. SPSS for windows, version 16.0.), and the significance level was considered at *p* < 0.05. In Table 2, which represents the demographic and clinical features, the qualitative variable gender is shown as a number and percentage. The *χ* ^2^ test analyzed the difference between the groups based on gender. The quantitative variables are presented as mean and standard division (*SD*), and the Mann–Whitney *U* test was used to detect the difference in the investigated variables between groups pairwise, including diabetic patients with and without obesity, controls with and without obesity, obese patients and controls, and nonobese patients and controls. Furthermore, Kruskal–Wallis was performed to examine variables among all four groups. In Table 3, the *χ* ^2^ test compared the distribution of the LPL S447X genotypes in patients and controls. Also, association between the LPL S447X variants with the risk of T2DM and obesity was investigated by a multivariable binary logistic regression model, which was adjusted for gender, age, lipid profile, and oxidative stress parameters.

**Table 1:** Characteristics of diabetic patients and controls

Variables	All T2DM patients (*n* = 132)	All controls (*n* = 118)	*p*‐value
Age (years)	53.6 ± 5.6	53 ± 4.90	0.35
BMI (kg/m^2^)	28.8 ± 6.3	28.2 ± 5.6	0.42
Waist circumference (cm)	97.4 ± 13	94.1 ± 11.6	0.036
Hip circumference (cm)	107.7 ± 14.5	106.2 ± 11.2	0.36
Waist/hip ratio	0.91 ± 0.16	0.88 ± 0.06	0.082
Wrist circumference (cm)	18 ± 2.2	17.1 ± 1.6	<0.001
FBS (mg/dl)	202.7 ± 70.3	87.9 ± 11.6	<0.001
Cholesterol (mg/dl)	189 ± 43.7	174.9 ± 26.9	0.002
TG (mg/dl)	184.2 ± 143.3	116.1 ± 44.6	<0.001
HDL‐C (mg/dl)	46.1 ± 17.5	49.1 ± 14.2	0.14
LDL‐C (mg/dl)	122.2 ± 34	109.7 ± 22.1	0.001
TAC (nmol/ml	3.1 ± 0.66	3.3 ± 0.73	0.02
TOS (nmol/ml)	14.3 ± 3.8	11.2 ± 2.8	<0.001
MDA (nmol/ml)	8.4 ± 3.9	5.7 ± 3.3	<0.001

Abbreviations: BMI, body mass index; FBS, fasting blood sugar; HDL‐C, high‐density lipoprotein‐cholesterol; LDL‐C, low‐density lipoprotein‐cholesterol; MDA, malondialdehyde; TG, triglycerides; T2DM, type 2 diabetes mellitus.

**Table 2:** The demographic and biochemical characteristics of different studied groups

Variables	Total	DM patients (obese, *n* = 67)	DM patients (normal BMI, *n* = 65)	Controls (obese, *n* = 62)	Controls (normal BMI, *n* = 56)	*p*‐value
	*n* = 250					
Female (%)	129 (51)	40 (59.7)	28 (43.1)	39 (62.9)	22 (39.3)	0.01^a^
Age (years)	53.3 ± 5.3	53.1 ± 5.2 *p* = 0.42^b^; *p* = 0.46^c^	54.1 ± 5.9	52.4 ± 4.5 *p* = 0.22^d^; *p* = 0.76^e^	53.6 ± 5.2	0.48^f^
BMI (kg/m^2^)	28.6 ± 6	34.2 ± 3.9 *p* < 0.001^b^; *p* = 0.05^c^	23.3 ± 1.7	32.9 ± 3.2 *p* < 0.001^d^; *p* = 0.88^e^	23.1 ± 2.1	<0.001^f^
Waist circumference (cm)	95.8 ± 12.5	106.7 ± 8.7 *p* < 0.001^b^; *p* = 0.001^c^	87.76 ± 9.07	101.2 ± 9.7 *p* < 0.001^d^; *p* = 0.30^e^	86.3 ± 7.9	<0.001^f^
Hip circumference (cm)	107 ± 13.1	117.3 ± 12.6 *p* < 0.001^b^; *p* = 0.005^c^	97.7 ± 8.2	114.5 ± 8.2 *p* < 0.001^d^; *p* = 0.29^e^	97 ± 5.5	<0.001^f^
Waist/hip circumference ratio	0.90 ± 0.12	0.92 ± 0.20 *p* = 0.47^b^; *p* = 0.13^c^	0.90 ± 0.11	0.88 ± 0.07 *p* = 0.53^d^; *p* = 0.85^e^	0.88 ± 0.05	0.47
Wrist circumferenc (cm)	17.6 ± 2	19.3 ± 2 *p* < 0.001^b^; *p* < 0.001^c^	16.7 ± 1.4	17.7 ± 1.3 *p* < 0.001^d^; *p* = 0.30^e^	16.4 ± 1.6	<0.001^f^
FBS (mg/dl)	148.52 ± 77.20	203.2 ± 61.1 *p* = 0.68^b^; *p* < 0.001^c^	202.2 ± 79.1	90.1 ± 12.6 *p* = 0.06^d^; *p* < 0.001^e^	85.5 ± 10.1	<0.001^f^
Cholesterol (mg/dl)	182.4 ± 37.4	194.8 ± 50.8 *p* = 0.15^b^; *p* = 0.15^c^	183.1 ± 34.3	178.4 ± 27.6 *p* = 0.01^d^; *p* = 0.10^e^	171.1 ± 25.9	<0.01^f^
TG (mg/dl)	152.1 ± 113.6	190.1 ± 98.6 *p* = 0.01^b^; *p* < 0.001^c^	178.2 ± 178.7	126.9 ± 44.4 *p* = 0.004^d^; *p* = 0.001^e^	104.1 ± 42.1	<0.001^f^
HDL‐C (mg/dl)	47.5 ± 16	48.3 ± 20.7 *p* = 0.20^b^; *p* = 0.76^c^	43.8 ± 13	47.4 ± 13.9 *p* = 0.09^d^; *p* = 0.002^e^	50.9 ± 14.3	0.02^f^
LDL‐C (mg/dl)	116.3 ± 29.6	123.7 ± 34.2 *p* = 0.82^b^; *p* = 0.02^c^	120.69 ± 34.05	111.2 ± 22.2 *p* = 0.33^d^; *p* = 0.01^e^	108 ± 22.1	<0.009^f^
TAC (nmol/ml)	3.1 ± 0.73	3 ± 0.61 *p* = 0.90^b^; *p* = 0.29^c^	3.1 ± 0.72	3.3 ± 0.78 *p* = 0.69^d^; *p* = 0.69^e^	3.2 ± 0.67	0.73^f^
TOS (nmol/ml)	12.9 ± 37.3	16.1 ± 3.4 *p* < 0.001^b^; *p* < 0.001^c^	12.5 ± 3.4	10.4 ± 2.3 *p* = 0.001^d^; *p* = 0.59^e^	12.2 ± 3.1	<0.001^f^
MDA (nmol/ml)	7.2 ± 4.2	9.8 ± 3.2 *p* < 0.001^b^; *p* < 0.001^c^	6.9 ± 4	4.8 ± 2.4 *p* = 0.009^d^; *p* = 0.95^e^	6.7 ± 3.8	<0.001^f^

*Note*: The gender variable is shown as a number and percentage. The quantitative variables are represented as the mean and *SD*.

Abbreviations: BMI, body mass index; DM, diabetes mellitus; FBS, fasting blood sugar; HDL‐C, high‐density lipoprotein‐cholesterol; LDL‐C, low‐density lipoprotein‐cholesterol; MDA, malondialdehyde; TG, triglycerides.

^a^ Comparing between groups (patients and controls) by *χ* ^2^ test.

^b^ Comparing between DM patients (obese and normal BMI) by Mann–Whitney *U* test.

^c^ Comparing between obese groups (DM patients and controls) Mann–Whitney *U* test.

^d^ Comparing between controls (obese and normal BMI) by Mann–Whitney *U* test.

^e^ Comparing between nonobese groups (DM patients and controls) by Mann–Whitney *U* test.

^f^ Comparing between groups (patients and controls) by Kruskal–Wallis test.

## 3 RESULTS

Anthropometric and biochemical parameters comparing all T2DM patients and all controls are demonstrated in Table 1. Significantly higher levels of waist and wrist circumference, FBS, total cholesterol, LDL‐C, TOS, and MDA, and significantly lower levels of HDL‐C and TAC were detected in patients compared to controls (Table 1). As given in Table 2. the mean age of the study population was 53.3 ± 5.3 years, and there was no difference between the study groups according to the age. Based on the *χ* ^2^ test, the female population was significantly higher than the male population in the four study groups (*p* = 0.01). The anthropometric assessment showed a significant difference between all groups, T2DM with and without obesity, T2DM and controls, obese patients and controls. Only normal BMI group of T2DM patients and controls had an insignificant difference regarding anthropometric parameters (Table 2). In addition, the waist‐to‐hip circumference ratio (WHR) was not significantly different comparing groups. The WHR in both sexes were compared separately. According to the WHO criteria (WHR of 0.90 or higher in men and 0.85 or more in women are considered for defining obesity)^15^ both men and women were obese in all studied groups. In males there were WHR of 0.94 ± 0.06, 0.91 ± 0.11, 0.93 ± 0.06, and 0.91 ± 0.05 in obese patients, normal BMI patients, obese controls and normal BMI controls, respectively. In women the WHR levels of 0.91 ± 0.25, 0.88 ± 0.1, 0.85 ± 0.06, and 0.85 ± 0.05 in obese patients, normal BMI patients, obese controls and normal BMI controls, respectively were detected.

Obese T2DM patients had the highest levels of cholesterol, but in the pairwise examination, the statistically significant difference was found in controls with and without obesity (*p* = 0.01). Normal BMI diabetic patients had significantly higher LDL‐C level (120.7 ± 34.1 mg/dl) than normal BMI controls (108 ± 22.1 mg/dl, *p* = 0.01). However, the mean HDL‐C level was significantly different comparing obese diabetic patients and normal BMI controls, and normal BMI individuals had the highest HDL‐C level (50.9 ± 14.3 mg/dl). The TG level was significantly lower in normal BMI individuals than in obese individuals (104.1 ± 42.1 vs. 126.9 ± 44.4 mg/dl, *p* = 0.004). However, the TOS level was considerably higher in the first group (Table 2).

The TAC level was compared between obese T2DM patients (3 ± 0.7 nmol/ml) and obese controls (3.3 ± 0.8 nmol/ml) that was significantly different (*p* = 0.03), and obese diabetic patients had a lower level of TAC than nondiabetic obese controls. The concentration of TOS showed a lower level in nondiabetic controls compared to the patient groups. Furthermore, there was a significant difference between patients with and without obesity. Obese‐diabetic patients had the highest TOS concentration (160.7 ± 33.7 nmol/ml, *p* < 0.001) dramatically. Also, the same result was observed in the MDA parameter that obese‐diabetic patients had the highest MDA level than the other groups of the study (Table 2).

Table 3 represents the frequency of the LPL S447X variants in various genetic models. In codominant model a significant difference in the frequency of the SX genotype between obese patients (3%) and obese controls (12.9%, *χ* ^2^ = 4.32; *p* = 0.038) was detected. Also, considering codominant model in comparison between all diabetic patients and all controls it was detected that in the presence of the SX genotype compared to the SS genotype the risk of T2DM reduced by 74% (odds ratio [OR] = 0.26, 95% confidence interval [CI]: 0.082–0.82, *p* = 0.022). In over dominant model comparing all diabetic patients with all controls indicated that in the presence of the SX genotype compared to the SS + XX genotype the risk of T2DM reduced by 75% (OR = 0.25, 95% CI: 0.08–0.8, *p* = 0.019).

**Table 3:** Distribution of LPL S447X genotypes and alleles in patients and controls

LPL (S447X)	T2DM patients (obese)	T2DM patients (normal BMI)	Controls (obese)	Controls (normal BMI)	All T2DM patients	All controls
					*n* = 132	*n* = 118
	*n* (%)	*n* (%)	*n* (%)	*n* (%)	*n* (%)	*n* (%)
**Genotype**						
**Codominant**						
SS	64 (95.5)	60 (92.3)	54 (87.1)	51 (91.1)	124 (94)	105 (89)
SX	2 (3)	2 (3.1)	8 (12.9)	5 (8.9)	4 (3)	13 (11)
XX	1 (1.5)	3 (4.6)	0 (0.0)	0 (0.0)	4 (3)	0 (0)
**Over dominant**						
SX	2 (3)	2 (3.1)	8 (12.9)	5 (8.9)	4 (3)	13 (11)
SS + XX	65 (97)	63 (96.9)	54 (87.1)	51 (91.1)	128 (97)	105 (89)
**Dominant**						
SS	64 (95.5)	60 (92.3)	54 (87.1)	51 (91.1)	124 (94)	105 (89)
SX + XX	3 (4.5)	5 (7.7)	8 (12.9)	5 (8.9)	8 (6)	13 (11)
**Recessive**						
XX	1 (1.5)	3 (4.6)	0 (0)	(0)	4 (3)	0 (0)
SS + SX	66 (98.5)	62 (95.4)	62 (100)	56 (100)	128 (97)	108 (100)
**Allele**						
S	130 (97)	122 (93.8)	116 (93.5)	107 (95.5)	252 (95.5)	223 (94.5)
X	4 (3)	8 (6.2)	8 (6.5)	5 (4.5)	12 (4.5)	13 (5.5)

*Note*: *χ* ^2^ = 12.4, *p* = 0.054 comparing three genotypes in codominant model between four groups of patients and controls. χ^2^ = 4.32, *p* = 0.038, OR = 0.21 (95% CI: 0.04–1.04, *p* = 0.055) comparing SS with SX genotype in codominant model between obese diabetic patients and obese controls.

*χ* ^2^ = 7, *p* = 0.07 comparing SX with SS + XX genotype in over dominant model between four groups of patients and controls.

*χ* ^2^ = 6.3, *p* = 0.012, OR = 0.25 (95% CI: 0.08–0.8, *p* = 0.019) comparing SX with SS + XX genotype in over dominant model all patients and all controls.

*χ* ^2^ = 9.6, *p* = 0.008 comparing three genotypes in codominant model between all patients and all controls. *χ* ^2^ = 5.9, *p* = 0.015, OR = 0.26 (95% CI: 0.082–0.82, *p* = 0.022) comparing SX with SS genotype in codominant model between all patients and all controls.

*χ* ^2^ = 1.99, *p* = 0.15 comparing SS with SX + XX genotype in dominant model between all patients and all controls.

*χ* ^2^ = 3, *p* = 0.38 comparing SS with SX + XX genotype in dominant model four groups of patients and controls.

*χ* ^2^ = 3.63, *p* = 0.057 comparing XX with SS + SX genotype in recessive model between all patients and all controls.

*χ* ^2^ = 5.7, *p* = 0.13 comparing XX with SS + SX genotype in recessive model between four groups of patients and controls.

*χ* ^2^ = 2.1, *p* = 0.54 comparing both alleles between four groups of patients and controls.

*χ* ^2^ = 0.24, *p* = 0.62 comparing both alleles between all patients and all controls.

Abbreviations: BMI, body mass index; CI, confidence interval; LPL, lipoprotein lipase; OR, odds ratio; T2DM, type 2 diabetes mellitus.

Also in the assessment of anthropometric, lipid profile, and oxidative stress parameters according to the LPL S447X variants in both codominant (SX vs. SS) and over dominant models (SX vs. SS + XX) in all individuals the SX genotype was associated with significantly lower levels of FBS, and TOS (Table 4).

**Table 4:** Comparing lipid profile, anthropometrics, and oxidative stress parameters based on LPL genotypes in all individuals

Variables	SS, *n* = 229 (mean ± *SD*)	SX, *n* = 17 (mean ± *SD*)	XX = 4 (mean ± *SD*)	SS + XX, *n* = 233^a^
BMI (kg/m^2^)	28.6 ± 5.9	28.7 ± 6.5	25 ± 3.7	28.5 ± 5.9
		*p* = 0.86	*p* = 0.13	*p* = 0.82
Waist circumference (cm)	95.9 ± 12.4	96.9 ± 13.8	90.5 ± 4	95.8 ± 12.3
		*p* = 0.77	*p* = 0.27	*p* = 0.74
Hip circumference (cm)	107 ± 13.2	108.3 ± 13.1	102.8 ± 6.7	106.9 ± 13.1
		*p* = 0.72	*p* = 0.45	*p* = 0.70
Waist/hip circumference ratio	0.90 ± 0.13	0.89 ± 0.07	0.88 ± 0.05	0.90 ± 0.13
		*p* = 0.77	*p* = 0.73	*p* = 0.77
Wrist circumference (cm)	17.5 ± 1.96	17.9 ± 2	17 ± 0.81	17.5 ± 1.9
		*p* = 0.48	*p* = 0.49	*p* = 0.47
FBS (mg/dl)	151.4 ± 78.7	107.1 ± 47.1	160 ± 46.7	151.5 ± 78.2
		*p* = 0.02	*p* = 0.97	*p* = 0.01
Cholesterol (mg/dl)	182.4 ± 38.3	179.2 ± 25.1	191.8 ± 24.2	182.6 ± 38.1
		*p* = 0.85	*p* = 0.47	*p* = 0.61
TG (mg/dl)	154.2 ± 117.1	118 ± 44.6	174.5 ± 107.3	154.5 ± 116.7
		*p* = 0.25	*p* = 0.65	*p* = 0.24
HDL‐C (mg/dl)	47.34 ± 16.3	47.6 ± 10.7	55.8 ± 20.7	47.5 ± 16.4
		*p* = 0.63	*p* = 0.38	*p* = 0.97
LDL‐C (mg/dl)	115.9 ± 29.2	113.2 ± 21.1	153.8 ± 61.8	116.5 ± 30.2
		*p* = 0.82	*p* = 0.15	*p* = 0.54
TAC (nmol/ml)	3.2 ± 0.66	3.2 ± 0.87	3.1 ± 0.35	3.2 ± 0.66
		*p* = 0.92	*p* = 0.94	*p* = 0.74
TOS (nmol/ml)	13.0 ± 3.7	11 ± 3.5	11.8 ± 4.4	13 ± 3.7
		*p* = 0.02	*p* = 0.31	*p* = 0.02
MDA (nmol/ml)	7.3 ± 4.2	7.2 ± 4.5	5.2 ± 1.9	7.1 ± 3.8
		*p* = 0.79	*p* = 0.36	*p* = 0.98

Abbreviations: BMI, body mass index; FBS, fasting blood sugar; HDL‐C, high‐density lipoprotein‐cholesterol; LDL‐C, low‐density lipoprotein‐cholesterol; LPL, lipoprotein lipase; MDA, malondialdehyde; TAC, total antioxidant capacity; TG, triglycerides; TOS, total oxidative status.

^a^ Compared to SX.

Table 5 represents the binary multivariable logistic regression analysis of the LPL S447X variants compared with the SS genotype as a reference genotype that adjusted for gender, age, anthropometric, lipid profile, and oxidative stress parameters. The presence of the SX genotype reduced the risk of T2DM by 79% (OR = 0.21; 95% CI: 0.05–0.81, *p* = 0.03) and obesity by 39% (OR = 0.61; 95% CI: 0.07–4.90, *p* = 0.6).

**Table 5:** Odds ratio (ORs) and 95% CI of the LPL genotypes and T2DM and obesity

LPL (S447X) genotypes	T2DM OR (95% CI)	*p*‐value	Obesity OR (95% CI)	*p‐*value
SS	Reference	–	Reference	–
SX	0.21 (0.05–0.81)	0.03	0.61 (0.07–4.90)	0.6
XX	–	–	0.31 (0.01–2.89)	0.7

*Note*: Model adjusted for gender, anthropometric, lipid profiles and oxidative stress parameters.

Abbreviations: CI, confidence interval; LPL, lipoprotein lipase; OR, odds ratio; T2DM, type 2 diabetes mellitus.

Considering all individuals, the HDL‐C level was significantly higher comparing all individuals with WHR < 0.90 (*n* = 115, 51.3 ± 17.9 mg/dl, *p* < 0.001) than those with WHR ≥ 0.90 (*n* = 125, 44 ± 13.2 mg/dl). The same pattern was observed considering obese patients (*n* = 29, 54.9 ± 27.3 vs. *n* = 38, 43.3 ± 12 mg/dl, *p* = 0.044) and obese controls (*n* = 32, 50.3 ± 15.1 vs. *n* = 26, 43 ± 10.6 mg/dl, *p* = 0.035), separately.

## 4 DISCUSSION

Predictions reveal that by 2045, the population with DM will reach 629 million people globally. Despite the treatments that help reduce blood glucose and lipid, patients with diabetes are associated with various macro‐ and micro‐vascular complications such as CVD and kidney dysfunction, and the mortality rate is alarmingly increasing among these patients.^1^, ^2^ Several environmental parameters, such as diet, obesity, and dyslipidemia, have been determined as risk factors for diabetes.^1^, ^6^ Nonetheless, the exact pathogenesis of the disease is not fully known.^2^ Therefore, figuring out the interaction between genetic factors (family history) and other metabolic factors such as dyslipidemia can help unravel the pathogenesis pathway of diabetes.^6^, ^14^ The current study examined the association of the LPL S447X polymorphism with T2DM and obesity in the Kurdish population of Iraq. In addition, the relation between the LPL S447X variants with anthropometric, lipid profile and oxidative stress parameters was assessed. Comparing all diabetic patients with all controls demonstrated significantly increased levels of anthropometric parameters (waist and wrist circumference), abnormal lipid profile and increased oxidative stress in patients. The initial evaluation of the four groups of our study showed a significantly highest amount of the anthropometric indices in obese diabetic patients. Also, the obese diabetic group, compared to the other groups, even normal BMI diabetic patients, had a higher level of oxidative stress parameters (TOS and MAD) and a lower level of TAC. Obesity induces oxidative stress via chronic inflammation, production of reactive oxygen species, and subsequently lipid peroxidation, and decreasing antioxidant capacity that are related to diabetes progression.^16^ According to the obtained results of the present study, we found that obese diabetic patients had statistically significant higher serum levels of TG, Chol, and LDL‐C than obese controls. Also, examination of lipid profile among normal BMI diabetic patients and normal BMI controls indicated that diabetic individuals had significantly higher levels of TG, Chol, and LDL‐C and lower level of HDL‐C.

In the analysis of LPL S447X variants between groups, results indicated that all individuals with the SX genotype compared to wild genotype of SS in codominant model and also compared to combined genotype of SS + XX in over dominant model had significantly lower levels of FBS, and TOS. Evidence expressed hypertriglyceridemia as common dyslipidemia in DM. Hypertriglyceridemia, or hepatic very low‐density lipoprotein‐TG secretion, a phenomenon not observed in T1DM, occurs due to imbalanced glucose metabolism and insulin resistance in T2DM. Furthermore, TG levels were higher at baseline and postprandially in individuals with T2DM.^17^ Gavra and colleagues, through the study on T2DM, observed that the carriers of the LPL SX genotype had significantly more negative oral fat tolerance test response (TG postprandial).^18^ However, the studies of Evans et al.^19^ and Shahzad et al.^10^ illustrated that the SX genotype of the LPL S447X was not associated with hyperlipidemia. The LPL S447X polymorphism cannot affect the catalytic activity of the LPL; however, this polymorphism might result in higher concentration and the activity of the LPL enzyme.^13^ Also, increased activity of the LPL improved the glucose metabolism.^20^ So, lower FBS levels found in the present study in carriers of the LPL SX genotype than individuals with wild genotype could be explained through benefit role of the polymorphism on glucose metabolism. Further, exposure to high level of plasma glucose increases the reactive oxygen species and consequently increased oxidative stress in hyperlipidemic individuals.^21^ In the present study lower TOS level in the presence of the LPL SX genotype could be due attributed to significantly lower levels of FBS in the presence of this genotype compared to the carriers of the wild genotype.

Regarding the LPL polymorphism, several studies investigated an association between the LPL S447X and T2DM, and the results imply the protective effect of the S447X polymorphism on T2DM incidence,^8^, ^22^, ^23^ as well as protection against diabetic nephropathy.^11^ In the present study the frequency of the SX genotype was higher in nondiabetic individuals than diabetic patients. The current study indicated the LPL SX genotype reduced the risk of T2DM. The protective role of the SX genotype compared to the SS genotype could be attributed to decreased FBS level and improved insulin sensitivity in the presence of elevated LPL concentration.^13^ Also, the LPL SX genotype had a protective role against obesity. In T2DM, LPL dysfunction or deficiency is commonly observed and is associated with atherosclerosis, Alzheimer's disease, obesity, and dyslipidemia.^23^ Nevertheless, studies on the LPL S447X polymorphism demonstrated that the polymorphism is related to increased LPL protein secretion and activity. The increased activity of LPL by decreasing TG and raising the HDL‐C levels can provide protection against T2DM and its complications, such as coronary artery diseases.^22^, ^24^ However, Hayne et al. did not find a higher LPL activity in the presence of the S447X polymorphism and suggested that the effect of the LPL S447X polymorphism as a cardioprotective phenotype is dependent on its role as a connecting bridge for uptake of the lipoproteins by hepatocyte.^25^

In the present study the HDL‐C level was significantly higher comparing individuals with WHR < 0.90 than those with WHR ≥ 0.90. The waist‐to‐hip circumference ratio assesses distribution or location of fat around the abdomen and hip. The higher value indicates high risk of CVD. It has been reported that this ratio affects the levels of total cholesterol and the ratio of LDL‐C to HDL‐C.^26^

## 5 CONCLUSION

The current study found that the obese diabetic patients had higher levels TG, Chol, LDL‐C, and lower level of HDL‐C than the control group (obese and normal BMI) and even nonobese diabetic individuals. The highest level of anthropometric indices observed in obese diabetic patients. Comparing various genotypes of the LPL S447X indicated the SX genotype significantly decreased FBS, and TOS levels. Also, our findings demonstrated that the LPL SX genotype decreased the risk of T2DM among patients from Kurdistan of Iraq. The result for obesity risk was similar and the SX genotype reduced the risk of obesity. Further, significantly higher level of HDL‐C was detected among individuals with WHR < 0.90 than those with WHR ≥ 0.90. Even though several studies investigated this polymorphism in the Asian population, there is a lack of evidence in the Middle East population. The present study was done in the Kurdish population of Iraq. Further studies on a large‐scale population and the evaluation of the interaction between various polymorphisms related to diabetic dyslipidemia could be helpful for understanding the pathogenesis of T2DM.

## AUTHOR CONTRIBUTIONS

**Gulshan Omar Ahmed**: conceptualization; data curation; investigation. **Zohreh Rahimi**: conceptualization; data curation; formal analysis; methodology; project administration; validation; writing – review & editing. **Maryam Kohsari**: formal analysis; writing – original draft; writing – review & editing. **Ebrahim Shakiba**: Validation. All authors have read and approved the final version of the manuscript.

## CONFLICTS OF INTEREST

The authors declare no conflicts of interest. Zohreh Rahimi is an Editorial Board member of Health Science Reports. and a co‐author of this article. To minimize bias, they were excluded from all editorial decision‐making related to the acceptance of this article for publication.

## ETHICS STATEMENT

The research followed the tenets of the Declaration of Helsinki. The Ethics Committee of Kermanshah University of Medical Sciences approved this study (IR.KUMS.REC. 1400.140). All participants entered the study after they were fully informed of the process and signed written consent.

## TRANSPARENCY STATEMENT

The lead author Zohreh Rahimi, Maryam Kohsari affirms that this manuscript is an honest, accurate, and transparent account of the study being reported; that no important aspects of the study have been omitted; and that any discrepancies from the study as planned (and, if relevant, registered) have been explained.

## Data Availability

The datasets used and/or analyzed during the current study are available from the corresponding author on reasonable request.
